# K121Q polymorphism in the *Ectonucleotide Pyrophosphatase/Phosphodiesterase* 1 gene is associated with acute kidney rejection

**DOI:** 10.1371/journal.pone.0219062

**Published:** 2019-07-18

**Authors:** Denise A. Sortica, Daisy Crispim, Andrea C. Bauer, Pamela S. Nique, Bruna B. Nicoletto, Ricieli P. Crestani, Jennifer T. Staehler, Roberto C. Manfro, Luis H. Canani

**Affiliations:** 1 Endocrine Division, Hospital de Clínicas de Porto Alegre, Porto Alegre, Rio Grande do Sul, Brazil; 2 Postgraduate Program in Medical Sciences: Endocrinology, Faculdade de Medicina, Universidade Federal do Rio Grande do Sul, Porto Alegre, Rio Grande do Sul, Brazil; 3 Division of Nephrology, Hospital de Clínicas de Porto Alegre, Porto Alegre, Rio Grande do Sul, Brazil; 4 Life Science Knowledge Area, Universidade de Caxias do Sul, Caxias do Sul, Rio Grande do Sul, Brazil, Nutrition Course, Área do Conhecimento de Ciências da Vida, Universidade de Caxias do Sul (UCS), Caxias do Sul, Brazil; Université Paris Descartes, FRANCE

## Abstract

The identification of risk factors for acute rejection (AR) may lead to strategies to improve success of kidney transplantation. Ectonucleotidases are ectoenzymes that hydrolyze extracellular nucleotides into nucleosides, modulating the purinergic signaling. Some members of the Ectonucleotidase family have been linked to transplant rejection processes. However, the association of Ectonucleotide Pyrophosphatase / Phosphodiesterase 1 (ENPP1) with AR has not yet been evaluated. The aim of this study was to evaluate the association between the K121Q polymorphism of *ENPP1* gene and AR in kidney transplant patients. We analyzed 449 subjects without AR and 98 with AR from a retrospective cohort of kidney transplant patients from Southern Brazil. K121Q polymorphism was genotyped using allelic discrimination-real-time PCR. Cox regression analysis was used to evaluate freedom of AR in kidney transplant patients according to genotypes. Q allele frequency was 17.6% in recipients without AR and 21.9% in those with AR (P = 0.209). Genotype frequencies of the K121Q polymorphism were in Hardy-Weinberg equilibrium in non-AR patients (P = 0.70). The Q/Q genotype (recessive model) was associated with AR (HR = 2.83, 95% CI 1.08–7.45; P = 0.034) after adjusting for confounders factors. Our findings suggest a novel association between the *ENPP1* 121Q/Q genotype and AR in kidney transplant recipients.

## Introduction

Kidney transplantation has become the treatment of choice for a substantial proportion of patients with end-stage renal disease. Transplantation has been shown to provide better quality of life and longer life expectancy than dialysis treatment [1, 2]. However, the survival rate in kidney transplant recipients is significantly lower than age-matched controls in the general population mainly due to recipient’s related factors, that include co-morbidities and duration of dialysis treatments, and transplant-related complications, such as infections, hypertension, diabetes, cancer, and acute rejection (AR) episodes [3, 4]. AR is correlated with progression to chronic allograft dysfunction, which is the most frequent cause of renal transplant failure [5].

AR is a potentially destructive allograft immune response that may occur at any time during the life span of a grafted organ. A number of factors are known to be associated with increased risk of AR, including recipient’s age, ethnicity, HLA sensitization and occurrence of delayed graft function (DGF). However, these factors alone may not fully account for all rejection episodes [6, 7]. Currently the gold standard for AR diagnosis is the allograft biopsy, an invasive procedure with related complications [8]. The development of noninvasive tools that help to predict the risk of AR might ultimately lead to strategies to improve patient and allograft outcomes [9, 10]. In this scenario, DNA polymorphisms that might be predictors of AR are worth to be thoroughly investigated [11].

Ectonucleotidases are ectoenzymes that hydrolyze extracellular nucleotides into nucleosides. They consist of four families, namely, ectonucleoside triphosphate diphosphohydrolases (NTPDases), ectonucleotide pyrophosphatase/phosphodiesterases (NPPs), ecto-5'-nucleotidases and alkaline phosphatases, and they are involved in the modulation of purinergic signaling [12]. Different ectonucleotidases, including NTPDase1 [13] and ecto-5’-nucleotidase, have been associated with chronic allograft rejection [14].

Ectonucleotide pyrophosphatase/phosphodiesterase 1 (ENPP1) belongs to the NPP family. This enzyme was first reported as a surface marker of B lymphocytes, being previously called plasma-cell differentiation antigen-1 (PC-1). ENPP1 is a cell membrane protein with an extracellular active site catalyzing the release of nucleoside 5-monophosphatase from nucleotides and their derivatives [15]. This enzyme is expressed in various tissues, including kidney, heart, brain, pancreatic islets, placenta, lung, salivary gland, epididymis, chondrocytes and lymphocytes [15]. It seems to be involved in immune system modulation [16, 17], possibly via degradation of extracellular adenosine triphosphate (ATP) and adenosine generation [18]. It has been shown that the K121Q polymorphism in the *ENPP1* gene is a risk factor for diabetes mellitus (DM) [15, 19] and diabetic kidney disease [20, 21], but its association with AR has not been evaluated.

Considering the potential involvement of different ectonucleotidases in transplant rejection, and a possible role of ENPP1 in immune modulation, we hypothesized that polymorphisms in the *ENPP1* gene might play a role in the AR process and, if so, could be used as predictor biomarkers of AR. Therefore, the aim of the present study was to evaluate the association between the *ENPP1* K121Q polymorphism and AR in a cohort of kidney transplant recipients.

## Materials and methods

### Study subjects

This nested case-control study was undertaken within a cohort of kidney transplant recipients from a tertiary teaching hospital in Southern Brazil. It was designed in accordance with STROBE and STREGA guidelines for reporting of genetic association studies [22, 23]. Six hundred and thirteen kidney transplant patients were initially recruited from 2002 to 2016. All recipients were followed-up for at least one year after transplantation. Among them, 501 (81.7%) patients were self-defined as white. Considering that the frequency of the *ENPP1* K121Q polymorphism differs between ethnic groups [24, 25], we excluded non-white subjects from the study. We also excluded patients without genotype data, biopsy proven acute antibody-mediated rejection and those who received a previous transplant, corresponding to 10.37% of the total group. Hence, the analyzed sample comprised 449 subjects. Among them, 98 patients had at least one AR episode (cases) and 351 patients did not present AR episodes during follow-up (control group) (S1 Fig).

### Study variables

Rejections that occurred within the first post-transplant year were diagnosed by an experienced transplant pathologist according to the Banff classification currently available [26–29]. Data were collected retrospectively from kidney transplant electronic records, and included: donor type [living or deceased (with brain death)], recipient and donor age at transplantation, recipient and donor gender, cold ischemia time, underlying kidney disease, number of pregnancies, number of blood transfusions, cytomegalovirus (CMV) and hepatitis C virus (HCV), renal replacement therapy modality, blood pressure (BP) previous to transplant, smoking habits, presence of DM or a family history of this disease, occurrence of DGF (defined by the requirement for hemodialysis in the first post-transplantation week), HLA mismatches, panel reactive antibody (PRA) class I/II, donor specific antibodies (DSA), induction immunosuppressive therapy, maintenance therapy, occurrence of post-transplant DM (PTDM) and time of AR diagnosis. Until 2006, HLA typing of donors and recipients were performed by PCR-SSP (polymerase chain reaction—sequence specific primers) technique [30], after that, the PCR-SSO (PCR—sequence specific oligonucleotide) technique was used [31]. The study was approved by the Ethics Committee of Hospital de Clínicas de Porto Alegre, and all subjects received adequate information about this study and gave their written informed consent.

### Genotyping of the *ENPP1* K121Q polymorphism

Peripheral blood samples were collected from all patients for DNA extraction and genotyping of the *ENPP1* K121Q polymorphism. DNA was extracted using a standardized salting-out procedure. Genotyping of the K121Q (A/C) polymorphism (rs1044498) in exon 4 of the *ENPP1* gene was performed using primers and probes contained in the Human Custom TaqMan Genotyping Assay 20x (Thermo Fisher Scientific Inc., Waltham, MA, USA). Primer and probe sequences used for genotyping were: 5-AGCCTCTGTGCCTGTTCAG-3’ (forward primer), 5’-ACACACAGAACTGTAGTTGATGCA-3’ (reverse primer), 5’-AGTCGCCCTTGTCCTT-3’ (VIC probe), and 5’-TCGCCCTGGTCCTT-3’ (FAM probe). All reactions were conducted in 96-well plates, in a total of 5 μl volume using 2 ng of genomic DNA, TaqMan Genotyping Master Mix 1x (Thermo Fisher Scientific Inc.) and Custom TaqMan Genotyping Assay 1x, and ran on the 7500 Fast Real-Time PCR System (Thermo Fisher Scientific Inc.).

### Statistical analyses

Allelic frequencies were determined by gene counting, and departures from the Hardy–Weinberg equilibrium (HWE) were verified using χ^2^ test. Allele and genotype frequencies were compared between groups of patients using χ^2^ tests. Clinical and laboratory characteristics were compared between groups by using unpaired Student’s t-test or χ^2^, as appropriate. Variables are shown as mean ± SD or absolute numbers (percentages). The magnitude of the associations with AR were estimated using odds ratios (ORs) with 95% confidence intervals (95% CI). Hazard ratio (HR) and 95% CI obtained from a Cox regression model was used to evaluate freedom of AR episodes in patients according to the presence of the 121Q/Q genotype (recessive model). The selection criteria for variables included in the Cox analysis were a well-established association with AR or P values < 0.10 obtained in the univariate Cox regression. In addition, logistic regression analysis was performed to test the independent association of the *ENPP1* K121Q polymorphism with AR, adjusting for the same covariates used in the Cox analysis. Results for which P values were less than 0.05 were considered statistically significant. Statistical analyses were performed using SPSS version 18.0 (SPSS, Chicago, IL).

## Results and discussion

### Sample description

Among the 449 renal transplant recipients included in this study, 98 presented biopsy proven acute cellular rejection and three hundred and fifty one patients did not have AR episodes (non-AR group). The median time for biopsy-proven AR was 12.5 days (2–329, minimum-maximum values). The main underlying diseases for CKD were: hypertension (22.8%), diabetic nephropathy (11.4%), primary glomerulonephritis (13.9%), and secondary glomerulonephritis (2%). Twenty one percent of the patients had an unknown underlying disease. Patient’s demographic and clinical characteristics are shown in Table 1. There were no significant differences between AR and non-AR groups regarding donor and recipient gender and age, number of blood transfusions CMV, HCV, number of pregnancies, renal replacement therapy modality, smoking habits, presence of DM, donor type, cold ischemia time, hypertension, HLA-A and B, DSA, and PRA class I or II. However, the mean of total HLA-mismatches was higher in the AR group than in the non-AR group (P = 0.011). Likewise, the frequency of patients with 2 HLA-DR mismatches was higher in the AR group than in the non-AR group (P = 0.008). Stratification of patients according to the two techniques used for HLA typing did not change the results. DGF occurred more frequently in the AR group compared to non-AR patients (P = 0.024). Moreover, a lower percentage of recipients with AR received antibody induction therapy with anti-thymocyte globulin (ATG) in comparison with the non-AR group (P = 0.004).

**Table 1:** Demographic and clinical characteristics of kidney transplant recipients classified by presence kidney acute rejection.

	AR (n = 98)*	Non-AR (n = 351)*	P values
Donor type (deceased), N (%)	64 (65.3)	253 (72.1)	0.240
Cold ischemia time, Yes (%)	14.04 ± 10.82	19.90 ± 74.92	0.441
Donor gender, N (%)	47 (53.4)	171 (53.4)	0.999
Donor age (years)	41.36 ± 16.05	42.43 ± 15.09	0.559
Recipient age (years)	42.41 ± 12.79	45.23 ± 12.78	0.054
Recipient gender, N (%)	61 (62.2)	210 (59.8)	0.752
Pregnancy, N (%)			
0	6 (16.2)	32 (22.7)	0.528
≥1	31 (83.8)	109 (77.3)	
Blood transfusion, N (%)			
0	49 (50.0)	180 (52.9)	0.690
≥1	49 (50.0)	160 (47.1)	
Positive CMV status, Yes (%)	24 (25.5)	57 (16.5)	0.080
Positive HCV status, Yes (%)	15 (9.1)	31 (15.8)	0.091
Renal replacement therapy, N (%)			
Hemodialysis	93 (94.9)	319 (90.9)	0.404
Peritoneal dialysis	4 (4.1)	22 (6.3)	
Preemptive transplant	1 (1.0)	10 (2.8)	
Smoking habits, Yes (%)	23 (23.5)	71 (20.2)	0.480
DM status, Yes (%)	9 (9.2)	47 (13.4)	0.346
DGF, Yes (%)	64 (65.3)	182 (51.9)	0.024
HLA-A mismatches (0/1/2)	12/48/36	50/170/122	0.857
HL-B mismatches (0/1/2)	8/47/41	59/157/126	0.094
HLA-DR mismatches (0/1/2)	25/40/31	122/155/61	0.008
HLA mismatches A/B/DR, mean	3.66	3.23	0.011
Hypertension, Yes (%)	72 (77.4)	282 (85.5)	0.090
PRA–Class I, N (%)	27 (45.8)	112 (48.7)	0.798
PRA–Class II, N (%)	25 (42.4)	93 (40.4)	0.903
DSA, Yes (%)	9 (10.1)	30 (9.3)	0.982
Antibody induction therapy, Yes (%)			
OKT3 or ATG or Basiliximab	58 (59.8)	260 (74.3)	0.008
ATG	13 (13.3)	99 (28.2)	0.004
OKT3	5 (5.1)	37 (10.5)	0.170
Basiliximab	44 (44.9)	154 (43.9)	0.948

Data are presented as mean ± SD or n (%).

* Unknown status for transfusion: n = 11, Delayed graft function (DGF): n = 1, HLA-A mismatches: n = 11, HLA-B mismatches: n = 11, HLA-DR mismatches: n = 15, HLA mismatches A/B/DR: n = 15, Hypertension: n = 27, Last panel reactive antibody Class I/II (PRA Class I/II): n = 161 (among them, 136 patients had ELISA-PRA test negative), Donor specific antibody (DSA): n = 38, OKT3: n = 1. Anti-thymocyte Globulin (ATG), Hepatitis C virus (HCV): n = 13, Smoking habits: n = 2, and Cytomegalovirus (CMV): n = 8.

Maintenance immunosuppression was achieved with prednisone, tacrolimus and mycophenolate for 269 patients (50.9%), 97 patients (18.4%) used prednisone, mycophenolate and cyclosporine, and the remaining patients (30.7%) received other combined therapies.

### Molecular analyses

Genotype frequencies were in HWE in controls (P = 0.70). The presence of Q/Q genotype was associated with AR assuming either additive (K/K *vs*. Q/Q; OR = 3.064; 95% CI 1.267–7.408; P = 0.020) or recessive (K/K+K/Q *vs*. Q/Q; OR = 3.210; 95% CI 1.343–7.673; P = 0.013) inheritance models (Table 2). The Q allele frequency was similar in AR patients compared to non-AR patients (21.9% *vs*. 17.6%, respectively; P = 0.209).

**Table 2:** Frequencies of the *ENPP1* K121Q polymorphism between kidney transplant patients with acute rejection (AR) and without acute rejection (non-AR).

*ENPP1* K121Q polymorphism	AR (n = 98)	Non-AR (n = 351)	P value	P value for additive model^a^	P value for dominant model^b^	P value for recessive model^c^
**Genotype**						
Q/Q K/Q K/K	10 (10.2)	12 (3.4)	0.019	0.020	0.835	0.013
	23 (23.5)	100 (28.5)				
	65 (66.3)	239 (68.1)				
**Allele**						
Q	0.219	0.176	0.209	-	-	-
K	0.780	0.823				

Data are shown as n (%) or proportion.

^a^Q/Q *vs*. K/K

^b^QQ/KQ *vs*. KK

^c^QQ *vs*. KQ/KK. P values were computed by χ^2^ tests for comparisons between groups.

**Fig 1:**
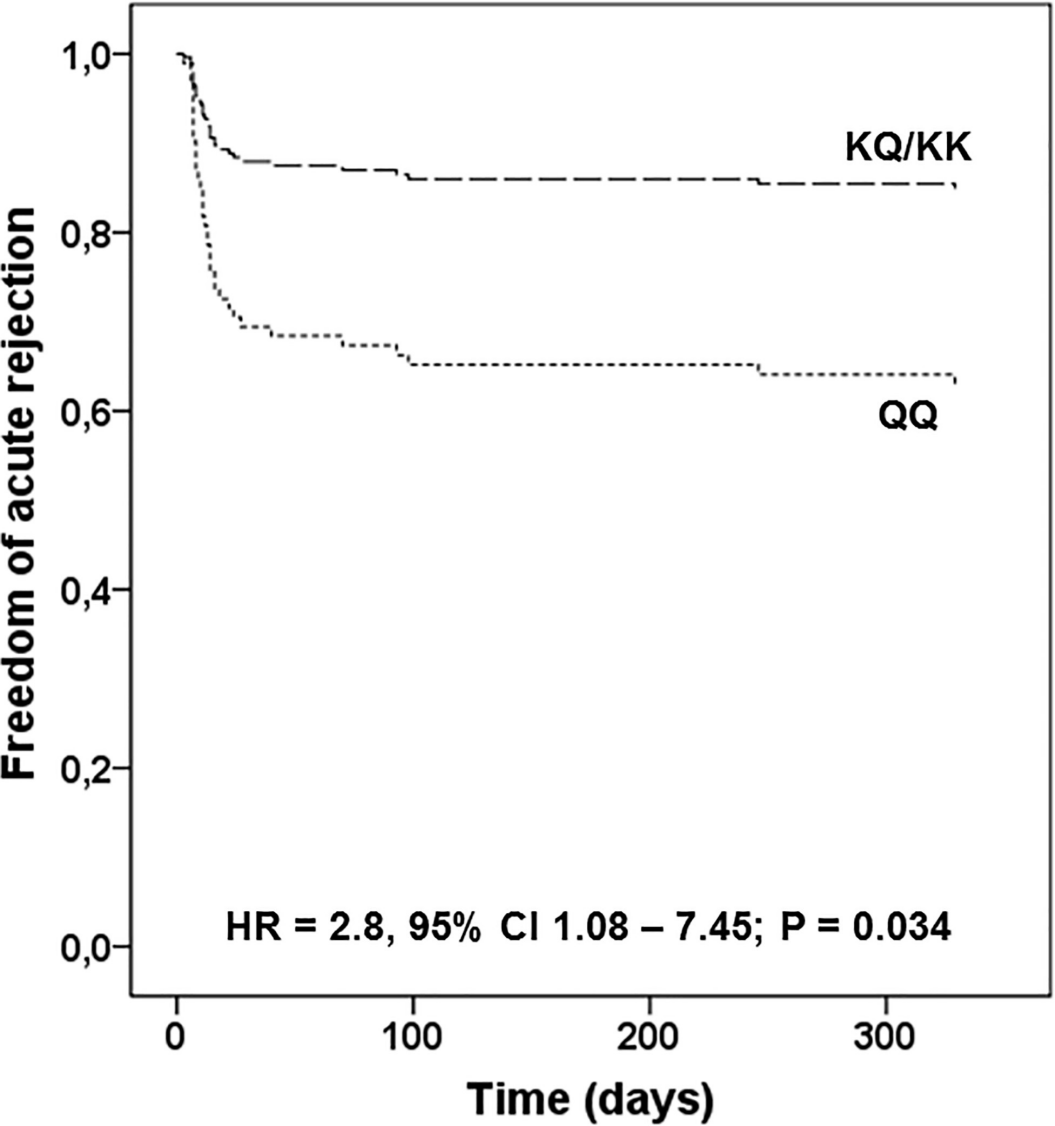
Cox regression analysis of the *ENPP1* K121Q polymorphism and acute rejection (AR) episodes in kidney transplant recipients. Adjusted for HLA-DR, pregnancies, blood transfusions, recipient age, delayed graft function, and induction therapy.

In a multivariable Cox regression analysis, the *ENPP1* 121Q allele remained associated with AR in the recessive model (HR = 2.838, 95% CI 1.080–7.457, P = 0.034; Fig 1) adjusting for HLA-DR mismatches, pregnancies, blood transfusions, recipient age, DGF, and induction therapy. In Table 3, we show HRs for each variable included in the Cox regression analysis. Among these variables, only DGF was associated with risk for AR (HR = 2.774, 95% CI 1.273–6.045, P = 0.010), while induction therapy was associated with protection against AR (HR = 0.345, 95% CI 0.158–0.755, P = 0.008). The independent association of the *ENPP1* 121Q/Q genotype with AR was confirmed by logistic regression analysis adjusting for HLA-DR mismatches, pregnancies, blood transfusions, recipient age, DGF, and induction therapy (OR = 6.41, 95% CI 1.46–28.19, P = 0.014; S1 Table).

**Table 3:** Multivariate Cox regression analysis of risk factors for AR.

Variable	HR	(95% CI)	P value
Receptors age	0.969	0.937–1.003	0.071
Pregnancy	1.434	0.548–3.758	0.463
Blood Transfusion	1.052	0.531–2.085	0.884
DGF	2.774	1.273–6.045	0.010
HLA-DR mismatches (1)	0.648	0.280–1.499	0.310
HLA-DR mismatches (2)	1.237	0.530–2.884	0.623
Q/Q genotype (recessive model)	2.838	1.080–7.457	0.034
Induction therapy	0.345	0.158–0.755	0.008

DGF, delayed graft function; HR, hazard ratio; CI, confidence interval.

## Conclusions

Here, we demonstrate, for the first time, the independent association of the *ENPP1* 121Q/Q genotype with AR in a cohort of white Brazilian kidney transplant patients.

**Fig 2:**
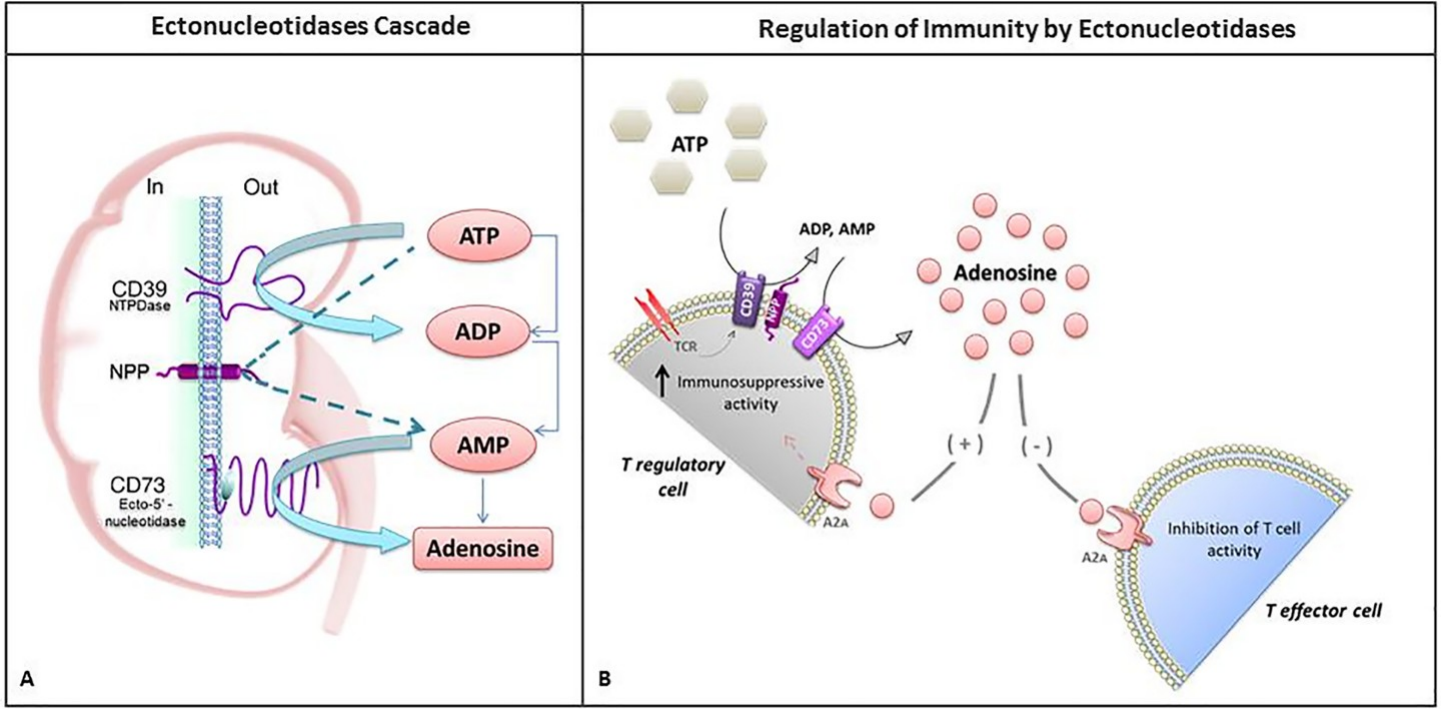
**(A) Ectonucleotidases cascade–**Members of the NTPDase (CD39) family are cell membrane enzymes that hydrolyze ATP into ADP as well as ADP into AMP through three different steps. In contrast, NPPs are able to degrade ATP and ADP into AMP in a single step, releasing AMP. In the final hydrolyzation step, the extracellular AMP can then be hydrolyzed to adenosine and inorganic phosphate (Pi) by the effect of Ecto-5′-nucleotidase (CD73). **(B) Regulation of immunity by ectonucleotidase cascade–**The occurrence of pathological insults, such as AR, activates T cell receptors (TCR) expressed in T regulatory cells (Treg), which induces CD39/CD73 activity leading to adenosine generation. Increased levels of extracellular adenosine promote immunosuppressive and anti-inflammatory activity in Treg cells. Also, through its receptor A2A in the T effector cell, adenosine suppresses T cell immunity by inhibiting activation of T effector cells. Thus, by employing different mechanisms on Treg and T effector cells, adenosine promotes an immunosuppressive effect.

Ectonucleotidases have been shown to modulate local immune responses by lymphocytes, with anti-inflammatory action that occurs, possibly, via degradation of the extracellular ATP and generation of adenosine [12, 18, 32, 33]. Members of the NTPDase (CD39) family are cell membrane enzymes that hydrolyze ATP into adenosine diphosphate (ADP) and ADP into adenosine monophosphate (AMP) in three different steps, releasing inorganic phosphate (Pi). Following that, ecto-5'-nucleotidases (CD73) dephosphorylate AMP into adenosine [34, 35]. In contrast, ENPP1 degrades ATP and ADP into AMP in a single step, releasing AMP along with pyrophosphate (PPi)[12]. In the final hydrolyzation step, the extracellular AMP can be hydrolyzed to adenosine and Pi by the effect of either ecto-5′-nucleotidase (CD73) or one of the four alkaline phosphatase isoforms [32] (Fig 2A).

NTPDase1 (CD39) and ecto-5'-nucleotidases (CD73), as major nucleotide metabolizing enzymes, are known to regulate immunity and inflammation and, possibly, to protect against hypoxic and ischemic tissue injuries [36]. Accordingly, CD39 and CD73 can be viewed as “immunological switches” that shift ATP-driven pro-inflammatory immune cell activity towards an adenosine mediated anti-inflammatory state [36]. Poelstra *et al* [37], studying a murine glomerulonephritis model, indicated that the ecto-5’-nucleotidase has an anti-inflammatory activity in glomerular cells. Therefore, it is conceivable to suppose that ENPP1 might play a role in the inflammatory process of organ transplant rejection. In this context, the 121Q allele that might lead to decreased ENPP1 activity could be a potential AR risk factor. Further experimental studies are needed in order to better clarify the putative role of ENPP1 in AR of organ allografts.

In order to speculate mechanistically on these findings it is important to mention that adenosine, released by ectonucleotidase activities (including ENPP1), is known to be an inhibitory mediator of T effector lymphocytes in various immune diseases [38–40]. CD39 and C73 are expressed on the surface of T regulatory (Treg) cells, converting ATP into adenosine, which acts as a substrate for Treg immunosuppressive and anti-inflammatory activities [41–43]. Therefore, it is possible that the presence of the *ENPP1* Q/Q genotype has an indirect negative effect on CD39 e CD73 activities, since mutated ENPP1 will generate less substrate for the other ectonucleotidases in the cascade. This might lead to increased activity of T effector lymphocytes and thus predispose to AR (Fig 2B). To confirm this hypothesis, the functional effect of the Q/Q genotype on ENPP1 activity in kidney transplant patients must be further explored.

It is established that HLA sensitizing events, such as pregnancies, blood transfusions, and prior transplants, might increase the risk of AR [44]. Likewise, donor and recipient characteristics, such as age and ethnicity also influence this outcome [45–47]. Also, HLA mismatches are a relevant risk factor for rejection [48, 49]. In our sample, only HLA-DR mismatches, occurrence of DGF and induction therapy with ATG were differently distributed between AR and non-AR groups. Noteworthy, the *ENPP1* K121Q polymorphism remained highly associated with AR after adjustment for HLA-DR mismatches, pregnancies, blood transfusions, recipient age, DGF, and induction therapy.

*ENPP1* K121Q polymorphism is differentially distributed across ethnicities [15, 20]. In this context, some studies showed that the Q allele has an increased prevalence among African-Brazilians [25] and other African descendent populations [50–52]. Based on this knowledge and also because the vast majority of our sample was comprised of white subjects, we evaluated only white subjects in the present study.

The present study has limitations to be acknowledged, including its retrospective design, and being a single center study without a confirmation cohort. However, we believe that the study of the K121Q polymorphism and its relationship with AR is worth evaluation in other populations in order to confirm or deny the present findings.

In conclusion, our findings support an association between the *ENPP1* K121Q polymorphism and AR in kidney transplant patients. The precise mechanisms behind this finding are uncertain and need to be further elucidated. Screening of this polymorphism may be useful to predict those patients (carriers of Q/Q genotype) more prone to experience rejection and, therefore, may need a more intense vigilance and perhaps more intense immunosuppressive therapy.

## Supporting information

S1 Fig Flowchart. Flowchart showing the strategy used to select patients for inclusion in the study.
(PDF)

S1 Table Multivariate logistic regression analysis of risk factors for AR. (DOCX)

S2 Table Minimal Data Set used for analyses. (XLS)
