# Supplementary material for: K121Q polymorphism in the Ectonucleotide Pyrophosphatase/Phosphodiesterase 1 gene is associated with acute kidney rejection
Source: PLoS One. 2019 Jul 18;14(7):e0219062. doi: 10.1371/journal.pone.0219062 (PMC6639061; doi:10.1371/journal.pone.0219062)
Supplement: S1 Fig — Flowchart showing the strategy used to select patients for inclusion in the study. (PDF) [file pone.0219062.s001.pdf]

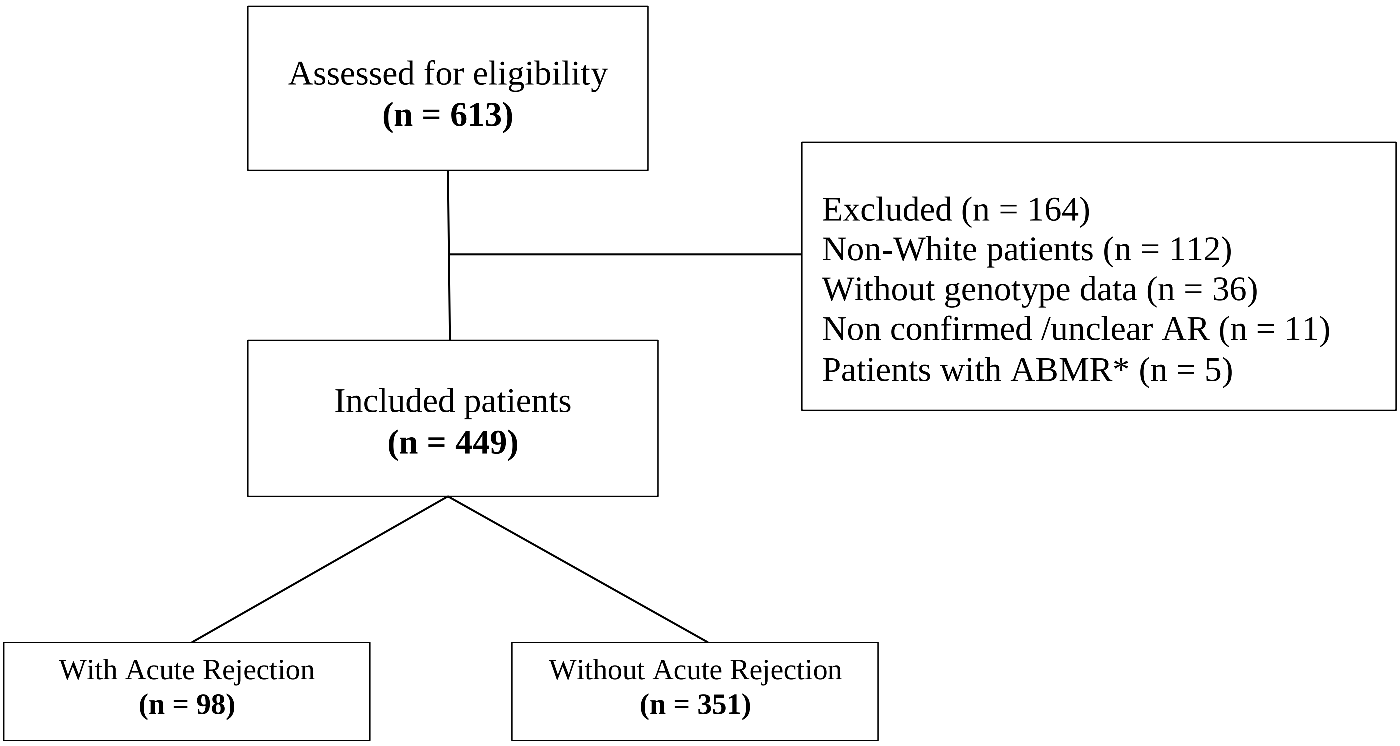

Supplementary Figure 1: Flowchart showing the strategy to select patients for inclusion in the study. *ABMR = antibody-mediated rejection.
