# Supplementary material for: K121Q polymorphism in the Ectonucleotide Pyrophosphatase/Phosphodiesterase 1 gene is associated with acute kidney rejection
Source: PLoS One. 2019 Jul 18;14(7):e0219062. doi: 10.1371/journal.pone.0219062 (PMC6639061; doi:10.1371/journal.pone.0219062)
Supplement: S1 Table — (DOCX) [file pone.0219062.s002.docx]

**S1_Table. Multivariate logistic regression analysis of risk factors for AR**.

| **Variable** | **OR** | **95% CI** | **P value** |
| --- | --- | --- | --- |
| Receptors age | 0.959 | 0.920 - 0.999 | 0.046 |
| Pregnancy | 1.601 | 0.515 - 4.978 | 0.416 |
| Blood Transfusion | 0.949 | 0.408 - 2.210 | 0.904 |
| DGF | 3.978 | 1.468 - 10.782 | 0.007 |
| HLA-DR mismatches (1) | 0.703 | 0.262– 1.888 | 0.484 |
| HLA-DR mismatches (2) | 1.447 | 0.509- 4.112 | 0.488 |
| Q/Q genotype (recessive model) | 6.418 | 1.461 - 28.196 | 0.014 |
| Induction therapy | 0.236 | 0.087 - 0.637 | 0.004 |

DGF, delayed graft function; ORs, odds ratios; CI, confidence interval.
